# Effect of laser acupuncture on adhesive small bowel obstruction: A prospective double-blind randomized controlled trial

**DOI:** 10.1097/MD.0000000000025035

**Published:** 2021-03-05

**Authors:** Chun-Han Shih, Ting-Min Hsieh, Bei-Yu Wu, Chun-Ting Liu

**Affiliations:** a Department of Chinese Medicine, Kaohsiung; b Department of Surgery, Division of Trauma, Kaohsiung Chang Gung Memorial Hospital and Chang Gung University College of Medicine; c Graduate Institute of Integrated Medicine, College of Chinese Medicine, Research Center for Chinese Medicine & Acupuncture, China Medical University, Taichung, Taiwan.

**Keywords:** acupuncture, adhesive small bowel obstruction, laser acupuncture

## Abstract

**Background:**

Adhesive small bowel obstruction (ASBO) is one of the most common complications and is a major cause of re-admission after intra-abdominal surgery. The initial management of patients with ASBO is nonoperative treatment such as nil per os and decompression using a nasogastric tube. However, the ideal management of ASBO remains controversial.

**Methods:**

This study will be a prospective, single-center, double-blind randomized controlled trial. Ninety two participants diagnosed with ASBO will be randomly assigned to either the verum or the sham laser acupuncture (SLA) group in a 1:1 ratio. All participants will undergo laser acupuncture (LA) or SLA once a day on 6 acupoints (LI4, PC6, ST25, ST36, CV4 and CV12) for 6 consecutive days after enrollment. The primary outcome measure will be the success rate of conservative treatment for ASBO. Secondary outcomes will be time to oral intake and length of hospital stay. The serum levels of lipase, amylase, cortisol, motilin, ghrelin, and intestinal fatty acid binding protein (I-FABP) will also be measured before intervention, on day 4, and on the day of discharge, respectively. Data will be analyzed by Chi-Squared test or t test between 2 groups.

**Objectives:**

The aim of this protocol is to investigate the clinical efficacy of LA on ASBO.

**Trial registration:**

ClinicalTrials.gov Identifier: NCT04318821. Registered on 24 March 2020.

## 1 Introduction

Adhesive small bowel obstruction (ASBO) is one of the most common complications and is a major cause of admission after intra-abdominal surgery. The incidence of ASBO following all types of abdominal surgery is 2.4%.^[1]^ Less commonly, the adhesions may form as a result of inflammatory conditions, intraperitoneal infection or abdominal trauma.^[2]^ Definitive confirmation of ASBO is made during operative treatment. In the initial 72 hours, nonoperative management for ASBO patients is appropriate unless there are signs of peritonitis, strangulation, or bowel ischemia.^[3,4]^ Approximately 70% to 90% of patients with ASBO respond to nonoperative treatment.^[3]^ Surgery intervention for adhesions should be considered for patients with repeated ASBO or unsuccessful conservative treatment, as any delay in surgical intervention is associated with higher mortality and complication rates if nonoperative management is unsuccessful after 3 days.^[4]^ To date, the ideal management of ASBO remains disputed. Therefore, the development of a new feasible treatment to increase the likelihood of successful nonoperative treatment and reduce the recurrence of ASBO is of great clinical significance.

Laser acupuncture (LA) combines the characteristics of traditional metal-needle acupuncture and low-level laser therapy. While acupuncture has been reported to promote the recovery time of postoperative ileus in patients with colorectal cancer,^[5]^ the application of LA for ASBO is very rare, and only 1 pre-clinical study has shown low level laser therapy to be effective in preventing intra-abdominal adhesions in rabbits without compromising the strength and healing of the abdominal wall.^[6]^ We assume that conventional treatment combined with LA will be effective for reducing the need for surgery through eliminating the symptoms of ASBO. The major aim of this study is to investigate the clinical benefits and the adverse events of LA in patients with ASBO. Furthermore, we want to explore the short-term effects or changes in several serum biomarkers associated with ASBO, including serum lipase, amylase, cortisol, motilin, ghrelin, and intestinal fatty acid binding protein (I-FABP). These serum biomarkers are associated with bowel movements and injuries, as explained in the discussion section.

## 2 Methods/design

### 2.1 Ethics approval

This protocol has been reviewed and approved by the Human Ethics Committee of the Chang Gung Medical Foundation Institutional Review Board (IRB no. 201901450A3; version 1 on October 28, 2019). The protocol identification number at https://clinicaltrials.gov is NCT04318821. This study will be conducted in accordance with the principles of the Declaration of Helsinki. Both verbal and written forms of detailed information about the trial will be provided before participation by trauma surgery physicians at Kaohsiung Chang Gung Memorial Hospital (KCGMH). All participants will provide voluntarily signed informed consent that has been approved by the ethics committee prior to enrollment. Personal information about potential and enrolled participants will be collected, shared, and maintained in an independent and secure storage space in order to protect confidentiality before, during, and after the trial. We will present the final results and submit them for publication in peer-reviewed journals. BYW and CTL will have access to the final trial dataset and disclosure of contractual agreements that limit such access for investigators.

### 2.2 Study design

**Figure 1:**
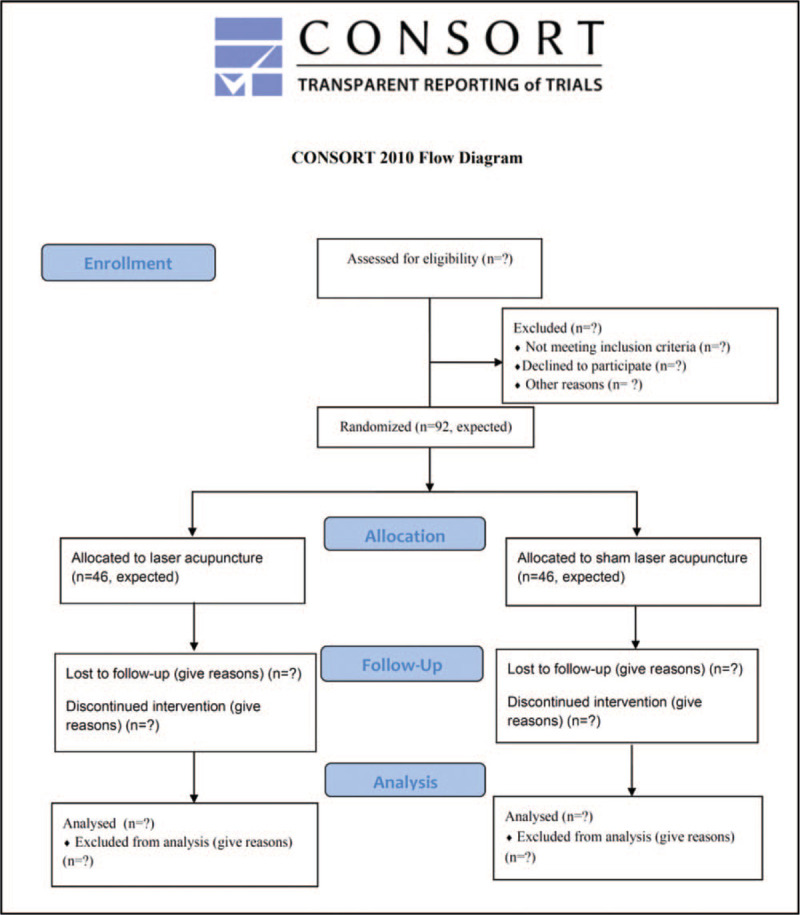
The flowchart of the trial.

This prospective, single-center, randomized, double-blind, controlled trial began at the Department of Trauma Surgery at the Kaohsiung Chang Gung Memorial Hospital (KCGMH) in South Taiwan from March 2020 and will continue until February 2022. Ninety two participants will be randomly assigned to the experimental group (LA plus conventional treatment, n = 46) or control group (sham LA [SLA] plus conventional treatment, n = 46). All participants will receive LA or sham LA after enrollment, once daily for 6 days, during hospitalization. The study design according to Consolidated Standards of Reporting Trials (CONSORT) 2010 is depicted in Figure 1.

### 2.3 Participants

The information of the trial will be provided by Trauma Surgery physicians at KCGMH. Participants’ eligibility in the study will be assessed by trauma surgeons. Written informed consents will be obtained for all participating patients before randomization. All patients with ASBO will be managed from the time of admission by the trauma surgery team, who have no information about the allocation results.

The study inclusion criteria are as follows:

1. patients aged 20 to 80 years;
2. patients with clinical symptoms and signs of mechanical obstruction, such as intermittent colicky abdominal pain, nausea with or without vomiting, abdominal distension, and absence of stools; and the results of plain abdominal radiographs performed with the patient upright or CT revealing dilated loops of the small intestine;
3. consent to participate in the study.

Participants with the following conditions will be excluded:

1. clear non-adhesive etiology of small bowel obstruction (e.g., tumor, hernia);
2. emergency surgical intervention before enrollment;
3. pregnancy;
4. local skin infection on the acupoints or limb amputees;
5. unwillingness to provide informed consent.

Participants will be dropped from the trial under the following conditions:

1. unstable vital signs and/or need for first aid during study period;
2. deterioration of clinical condition and determination of unsuitability to continue this study by medical staff;
3. voluntary decision by participants to withdraw from the trial at any time.

### 2.4 Sample size, blinding, and randomization

As no related studies on the effects of acupuncture or laser acupuncture for the treatment of ASBO have been published, we determined the necessary sample size based on a previous study of the effects of sesame oil on ASBO.^[7]^ The surgical rate was 12.9% in the experimental group and 48.5% in the control group. Anticipating a power of 95% (1-β = 0.95), statistical significance (α = 0.05) of 95%, and a dropout rate of 10%, a total of 92 participants will be required in this study, according to G∗power analysis.

All the participants will be informed that they will receive laser acupuncture in addition to the conventional treatment and assess the efficacy of laser acupuncture for ASBO. The laser acupuncture devices used in the LA and SLA groups will have the same appearance. The difference will be that no energy will be output during the intervention in the SLA group. The participants will be unable to recognize whether they are being treated or not because laser acupuncture involves low-intensity, non-thermal laser irradiation that it is hard to detect. Participants will be randomly allocated to either the LA or the SLA group in a 1:1 ratio using computer-generated random numbers. Randomization will be performed by an independent researcher with no involvement in the inclusion and exclusion process, treatment, or outcome assessments.

The participants, outcome assessors, and data analysts will be blinded to the allocation of interventions using labels A and B for the 2 groups until the trial is completed.

### 2.5 Interventions

All participants will receive conventional treatment, including nonoperative managements, initially, and perhaps further surgical intervention if indicated according to the Bologna guidelines of ASBO^[3]^ and the trauma gastrointestinal surgeons’ expertise. Therapeutic efficacy will be defined by the appearance of flatus/defecation, radiological improvement, relief of abdominal symptoms, decreased drainage volume of nasogastric decompression, disappearance of air-fluid levels, or reduced gas and fluid in bowel loops. The nasogastric tube will be removed when the drainage output decreases to less than 200 ml per day or flatus/defecation occurs. A liquid diet will be administered gradually, typically followed by a soft diet and then solid food as tolerated. Patients will be discharged when solid food is well tolerated. If the patient presents no improvement and progresses into strangulation, peritonitis, or bowel ischemia, surgery will be recommended.^[3]^

**Figure 2:**
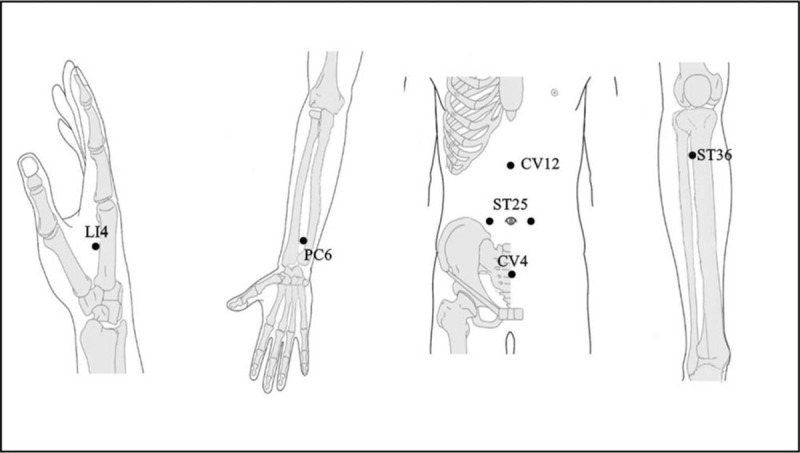
Acupoints used in the study: LI4, PC6, ST25, ST36, CV12, and CV4.

The participants in each group will undergo real LA or sham LA treatment once daily for 6 days, using a gallium aluminum arsenide Laser Pen (maximal power, 150 mW; wavelength, 810 nm; area of probe, 0.03 cm^2^; power density, 5 W/cm^2^; pulsed wave; and Bahr frequencies (B1: 599.5 Hz, B2: 1199 Hz, B3: 2398 Hz, B4: 4776 Hz, B5: 9552 Hz, B6: 19,104 Hz, and B7: 38,208 Hz]; RJ-Laser, Reimers & Janssen GmbH, Waldkirch, Germany). We will use the same acupoints and manipulations in both groups. The participants in the control group will receive SLA treatment without any laser output (no stimulation), while the participants in the experimental group will sequentially receive 0.375 J of energy at each of the following acupoints: LI4 (Hegu, B3), PC6 (Neiguan, B3), ST25 (Tianshu, B3), ST36 (Zusanli, B2), CV4 (Guanyuan), and CV12 (Zhongwan, B3) (Fig. 2). The laser treatment will be applied to each point for 5 seconds to deliver a total treatment dose of 4.5 J/cm^2^. All acupoints have been selected and localized according to the WHO Standardized Acupuncture Point Location guidelines.^[8]^ In all the subjects, the laser application will be performed by the same experienced physician, who has sufficient training and is a licensed Chinese medicine practitioner in Taiwan.

### 2.6 Outcome measurements

The primary outcome measurement will be the success of conservative treatment, which is defined as no need for nasogastric tube decompression or operative intervention. Secondary outcome measurements will include time to oral intake, length of hospital stay, and changes in serum amylase, lipase, cortisol, motilin, ghrelin, and I-FABP levels, which will be checked before the intervention, on the fourth day, and on the day of discharge, respectively. The reasons why participants are unable to complete the follow-up treatments or withdraw from the study, such as adverse events, suboptimal response to therapy, surgical intervention for ASBO, or refusal to receive intervention, will be recorded.

### 2.7 Statistical analysis

We will compare the results of the statistical analyses between the LA and the SLA groups. The data will be expressed as frequencies, percentages or mean ± standard deviation and analyzed by the Student *t* test or the Chi*-*Squared test. All analyses will be performed in SPSS software. Differences will be considered statistically significant at *P* < .05.

### 2.8 Data monitoring

No data monitoring committee (DMC) will be needed because laser acupuncture is a general practice and noninvasive intervention. Nevertheless, adverse events such as unexplained diarrhea, increased abdominal pain, and fainting during laser acupuncture treatment will be monitored during the trial period.

## 3 Discussion

Typically, non-invasive methods applied to confirm of ASBO include a history of previous episodes of bowel obstruction by adhesions, or exclusion of other causes of bowel obstruction by imaging.^[3]^ The initial nonoperative management of patients with ASBO during first 72 hours, such as nil per os and decompression using a nasogastric tube or long intestinal tube, is safe and appropriate unless there are signs of peritonitis, strangulation, bowel ischemia, or other signs of clinical deterioration.^[3,4]^ Previous research found that the average length of stay in hospitals for ASBO was 7.8 days, and the in-hospital mortality from ASBO was 2.5%.^[1]^ Overall, recurrence or readmission due to ASBO is common, occurring in about 20% of patients.^[9,10]^ Although the risk of recurrence is slightly lower after operative treatment,^[9]^ this may not be the reason for deciding whether or not to prescribe a surgical intervention. Currently, the use of surgery to treat ASBO is controversial because such a procedure may induce the formation of new adhesions, increase postoperative morbidity, and length of hospital stay, incur higher associated costs as compared with nonoperative treatment, and significantly reduce postoperative quality of life.^[3,10,11]^Therefore, it is of great significance to find a treatment that can improve the success rate of conservative treatment for ASBO.

Acupuncture therapy, one of the most popular forms of complementary and alternative medicine (CAM), is used for the treatment of a variety of conditions.^[12]^ LA is a type of photo biomodulation under the instruction of traditional Chinese medicine theory. It involves stimulation of traditional acupoints with low-intensity, non-thermal, noninvasive laser irradiation.^[13]^ LA has become more common among acupuncture practitioners in recent years for its minimal sensation, short duration of treatment, and minimal risks of infection, trauma, and bleeding complications.^[13]^ LA has been shown to inhibit formations of postoperative intra-abdominal adhesions in animal studies.^[14,15]^ However, as far as we know, no trials have examined the clinical efficacy of acupuncture or LA on ASBO. Thus, we designed this trial to investigate the benefits and adverse events of LA in patients with ASBO.

In addition to assessing the clinical symptoms of ASBO, we will also try to assess the changes in serum biomarkers associated with bowel movements and injury. Elevated lipase and amylase levels are usually the diagnostic base of acute pancreatitis. However, elevation of lipase and amylase levels is also observed in patients with small bowel obstruction.^[16]^ Elevated serum lipase is also a risk factor for higher serum creatinine, lactate, use of intensive care, and mortality.^[17]^ We assume that elevated serum lipase and amylase levels may have poor prognosis and prolong the length of hospital stay. Cortisol is a marker not only for diagnosing hypo- or hypercortisolism but also diseases of metabolism, inflammation, the cardiovascular system, and behavior.^[18,19]^ A flatter diurnal cortisol slope is associated with poorer health and may be a marker or a mechanism for disease etiology.^[20]^ We think cortisol can be treated as a stress response to inflammation and as a tool to assess the efficacy of treatment. Ghrelin and motilin are produced in the mucosal layer of the stomach and duodenum, respectively. They are hormones that can stimulate appetite and gastrointestinal motility.^[21]^Motilin is known to contract the gastrointestinal tract through activation of smooth muscle cells, local enteric neurons, and afferent terminals of vagus nerves. Ghrelin has multiple functions; in addition to modulating gastrointestinal motility, it is also involved in glucose metabolism, lipid metabolism, and endocrine functions.^[21]^ We think motilin and ghrelin can be used for evaluating the stimulative effect of gastrointestinal motility. I-FABP is involved in the uptake and transport of long chain fatty acids from the intestinal lumen.^[22]^ I-FABP is mainly expressed in the intestinal villi, where ischemic injury first occurs, so it might be an early and useful marker for mucosal compromise or injury of the intestine as well as systemic inflammatory response syndrome.^[23]^ Thus, we will use I-FABP as a diagnostic marker for evaluating gut wall integrity loss and inflammation.

In conclusion, the results of this study are expected to indicate the efficacy of LA as a nonoperative treatment in the management of ASBO in addition to conventional treatments.

## Acknowledgments

The authors thank the Biostatistics Center, Kaohsiung Chang Gung Memorial Hospital for statistics work.

## Author contributions

**Conceptualization:** Bei-Yu Wu, Chun-Ting Liu.

**Data curation:** Bei-Yu Wu.

**Formal analysis:** Bei-Yu Wu.

**Investigation:** Ting-Min Hsieh.

**Project administration:** Ting-Min Hsieh.

**Supervision:** Chun-Ting Liu.

**Writing – original draft:** Chun-Han Shih.

**Writing – review & editing:** Chun-Ting Liu.

## References

[1] Ten Broek RPG Issa Y van Santbrink EJP. Burden of adhesions in abdominal and pelvic surgery: systematic review and met-analysis. BMJ 2013;347:f5588–15588. 24092941 10.1136/bmj.f5588 PMC3789584

[2] Attard J-AP MacLean AR. Adhesive small bowel obstruction: epidemiology, biology and prevention. Can J Surg 2007;50:291–300. 17897517 PMC2386166

[3] Ten Broek RPG Krielen P Di Saverio S. Bologna guidelines for diagnosis and management of adhesive small bowel obstruction (ASBO): 2017 update of the evidence-based guidelines from the world society of emergency surgery ASBO working group. World J Emerg Surg 2018;13:1–3. 29946347 10.1186/s13017-018-0185-2 PMC6006983

[4] Keenan JE Turley RS McCoy CC. Trials of nonoperative management exceeding 3 days are associated with increased morbidity in patients undergoing surgery for uncomplicated adhesive small bowel obstruction. J Trauma Acute Care Surg 2014;76:1367–72. 24854302 10.1097/TA.0000000000000246

[5] Liu Y May BH Zhang AL. Acupuncture and related therapies for treatment of postoperative ileus in colorectal cancer: a systematic review and meta-analysis of randomized controlled trials. Evid Based Complement Alternat Med 2018;2018:3178472 1–18. 30151019 10.1155/2018/3178472 PMC6087601

[6] Teixeira ML Vasconcellos LS Oliveira TG. Prevention of abdominal adhesions and healing skin after peritoniectomy using low level laser. Lasers Surg Med 2015;47:817–23. 26415104 10.1002/lsm.22423

[7] Camporesi EM. Side effects of hyperbaric oxygen therapy. Undersea Hyperbaric Med 2014;41:253–7. 24984321

[8] World Health Organization. WHO standard acupuncture point locations in the Western Pacific Region. 2008;35–225.

[9] Behman R Nathens AB Mason S. Association of surgical intervention for adhesive small-bowel obstruction with the risk of recurrence. JAMA Surg 2019;154:413–20. 30698610 10.1001/jamasurg.2018.5248 PMC6537786

[10] Wessels LE Calvo RY Dunne CE. Outcomes in adhesive small bowel obstruction from a large statewide database: what to expect after nonoperative management. J Trauma Acute Care Surg 2019;86:651–7. 30907786 10.1097/TA.0000000000002196

[11] Catena F Di Saverio S Coccolini F. Adhesive small bowel adhesions obstruction: Evolutions in diagnosis,1; management and prevention. World J Gastrointest Surg 2016;8:222–31. 27022449 10.4240/wjgs.v8.i3.222 PMC4807323

[12] Ernst E. Acupuncture--a critical analysis. J Intern Med 2006;259:125–37. 16420542 10.1111/j.1365-2796.2005.01584.x

[13] Chon TY Mallory MJ Yang J. Laser acupuncture: a concise review. Med Acupunct 2019;31:164–8. 31297170 10.1089/acu.2019.1343 PMC6604908

[14] Du MH Luo HM Tian YJ. Electroacupuncture ST36 prevents postoperative intra-abdominal adhesions formation. J Surg Res 2015;195:89–98. 25619463 10.1016/j.jss.2014.12.043

[15] Zhang L Wang H Huang Z. Inhibiting effect of electroacupuncture at zusanli on early inflammatory factor levels formed by postoperative abdominal adhesions. Evid Based Complement Alternat Med 2014;2014:950326 1–5. 25197314 10.1155/2014/950326 PMC4145794

[16] Nereo Vettoretto GP Matheos Romessis Andrea Ferrari Bravo. Laparoscopy in afferent loop obstruction presenting as acute pancreatitis. J Soc Laparosc Robot Surg 2006;10:270–4. PMC3016119 16882437

[17] Fathima Z Kamil Faiz SM Mahak Saad. Prognostic value of serum lipase levels in patients with small bowel obstruction. Proc (Bayl Univ Med Cent) 2018;31:276–9. 29904287 10.1080/08998280.2018.1446637 PMC5997060

[18] Rogier A Quax LM Jan W Koper. Glucocorticoid sensitivity in health and disease. Nat Rev Endocrinol 2013;9:670–86. 24080732 10.1038/nrendo.2013.183

[19] Vincent L Wester EFCvR. Clinical applications of cortisol measurements in hair. Eur J Endocrinol 2015;173:M1–10. 25924811 10.1530/EJE-15-0313

[20] Emma K Adam MEQ Royette Tavernier. Diurnal cortisol slopes and mental and physical health outcomes: a systematic review and meta-analysis. Psycho Neuro Endocrinol 2017;83:25–41. 10.1016/j.psyneuen.2017.05.018 PMC5568897 28578301

[21] Kitazawa T Kaiya H. Regulation of gastrointestinal motility by motilin and ghrelin in vertebrates. Front Endocrinol 2019;10:278 1–17. 10.3389/fendo.2019.00278 PMC6533539 31156548

[22] Takeshi K YHK Kathryn Kiefer Rommel Morales. Circulating intestinal fatty acid-binding protein (I-FABP) levels in acute decompensated heart failure. Clin Biochem 2017;50:491–5. 28232029 10.1016/j.clinbiochem.2017.02.014 PMC5474350

[23] Maika Voth TL Borna R Ingo M. Is I-FABP not only a marker for the detection abdominal injury but also of hemorrhagic shock in severely injured trauma patients? World J Emerg Surg 2019;14:49 1–9. 31832083 10.1186/s13017-019-0267-9 PMC6868704

